# Spectrum of Genetic Changes in Patients with Non-Syndromic Hearing Impairment and Extremely High Carrier Frequency of *35delG GJB2* Mutation in Belarus

**DOI:** 10.1371/journal.pone.0036354

**Published:** 2012-05-02

**Authors:** Nina Danilenko, Elena Merkulava, Marina Siniauskaya, Olga Olejnik, Anastasia Levaya-Smaliak, Alena Kushniarevich, Andrey Shymkevich, Oleg Davydenko

**Affiliations:** 1 Institute of Genetics and Cytology, National Academy of Sciences, Minsk, Belarus; 2 Belarusian State Medical University, Minsk, Belarus; 3 Estonian Biocentre, Tartu, Estonia; Stanford University School of Medicine, United States of America

## Abstract

The genetic nature of sensorineural hearing loss (SNHL) has so far been studied for many ethnic groups in various parts of the world. The single-nucleotide guanine deletion (*35delG*) of the GJB2 gene coding for connexin 26 was shown to be the main genetic cause of autosomal recessive deafness among Europeans. Here we present the results of the first study of GJB2 and three mitochondrial mutations among two groups of Belarusian inhabitants: native people with normal hearing (757 persons) and 391 young patients with non-syndromic SNHL. We have found an extremely high carrier frequency of *35delG* GJB2 mutation in Belarus −5.7%. This point deletion has also been detected in 53% of the patients with SNHL. The 312del14 GJB2 was the second most common mutation in the Belarus patient cohort. Mitochondrial A1555G mt-RNR1 substitution was found in two SNHL patients (0.55%) but none were found in the population cohort. No individuals carried the A7445G mutation of mitochondrial mt-TS1. G7444A as well as T961G substitutions were detected in mitochondrial mt-RNR1 at a rate of about 1% both in the patient and population cohorts. A possible reason for Belarusians having the highest mutation carrier frequency in Europe *35delG* is discussed.

## Introduction

The genetic mechanisms of hearing loss have been extensively analyzed during the last two decades. Over 100 loci, both nuclear and mitochondrial, are already implicated in the development of this most common sensory disorder of humans [1]. The GJB gene family (GJB2, GJB3 and GJB6) encoding gene gap junction proteins – connexins – was found to be the main “storage place" of mutations causing non-syndromic sensorineural hearing loss (SNHL) [2]–[4]. More than 50% of cases of autosomal recessive non-syndromic hearing loss in many world populations are attributed to mutations in GJB2 gene encoding connexin 26 (OMIM: 121011) [5].

The most frequent GJB2 anomaly causing deafness in Europeans and white Americans is deletion of one guanine within the six-guanine string at the beginning of the second GJB2 exon (positions 30–35), the so-called *35delG* mutation (rs80338939) [6], [7]. Japanese people carry another recessive major GJB2 mutation, *235delC* (rs80338943); its prevalence in Japan exceeds 73% [8]. *V37I* (rs72474224) is frequently found among Taiwan natives [5] and *167delT* (rs80338942) in Ashkenazi Jews [9]. Besides the point mutations and small deletions, several large deletions were detected on the 13th chromosome within the region where GJB2 and GJB6 genes are positioned close to each other. These are the 309 kb (GJB6-D13S1830) and 232 kb deletions (GJB6-D13S1854) located more than 100 kb upstream of the transcriptional start sites of the GJB2 and GJB6 genes. Both deletions are frequently detected in compound heterozygosity with point *GJB2* mutations [10], [11].

The average carrier rate of *35delG* GJB2 mutation in Europe is about 2% [12]. In some Mediterranean countries (Italy (Sardinia) Greece and Malta) it exceeds 3%. A moderately high rate (4.5%) was reported for Estonia, one of the Baltic countries [3], [5]. For one of the Volga-middle Ural ethnic group, Mordvins, the *35delG* GJB2 carrier frequency is even higher (6.2%) [13]. All these values are surprisingly high and point either to the “founder effect" or selective advantage for heterozygotes, or both. Indeed, according to a recent publication [14], the epidermal thickness is significantly higher among European individuals heterozygous or homozygous for *35delG* compared to wild-type. These data stand in line with the previously reported fact that Africans who carry the mutant GJB2 allele *R134W* (rs80338948) have a thicker epidermis than the wild-type. *In vitro* studies have demonstrated that cells expressing the *R134W* allele are significantly less susceptible to cellular invasion of the enteric pathogen *Shigella flexneri* than wild-type cells [15], [16]. These data support the hypothesis that skin phenotype might counterbalance the evolutionary disadvantage caused by deafness [14].

Besides the nuclear genes, a proper set of mitochondrial (mt) genes is involved in the control of normal hearing [17], [18], [19]. Mutations of mitochondrial genes have some peculiarities: they are maternally inherited; they can be both in homo- and heteroplasmic states. Though definite mitochondrial point mutations and large deletions usually lead to syndromic deafness [20], [21], some point mutations, notably in mt-RNR1 and mt-TS1 (*tRNA^Ser^*) genes, may cause SNHL [17]. A certain range of mutations in the mt*-RNR1* gene (*A1555G, C1494T, T961G*) was found to be associated with aminoglycoside-induced deafness [22]. The incidence of these mutations varies also among different populations; so far nothing has been reported about Belarusian patients with SNHL.

As far as we know, the only data on the *35delG* GJB2 carrier rate among Belarusians (97 people with normal hearing) were published in 2010 [13]. The carrier frequency was reported to be more than 6%, the highest rate for European countries studied so far. Here we present, firstly, results of an extensive analysis of *35delG/*GJB2 carrier frequency among more than 750 modern Belarusians from six ethno geographic regions of Belarus. Secondly, we summarize the results of the analysis of GJB2 gene structural changes along with the 309 kb deletion truncating the neighboring GJB6 gene del(GJB6-D13S1830) in a large Belarus patient cohort with SNHL (391 patients). Finally, both the Belarus population and patient cohorts were screened for four mitochondrial mutations associated with deafness.

We provide here the genetic evidence that the carrier frequency of the *35delG* deafness mutation in the Belarus population is the highest compared to other countries. The same GJB2 defect is the main reason for SNHL in Belarus patients. Additionally our study reveals that the second most common GJB2 mutation in the Belarus patient cohort with SNHL is the *312del14* deletion.

## Results and Discussion

### Carrier rate of 35delG GJB2 mutation in Belarus

**Figure 1:**
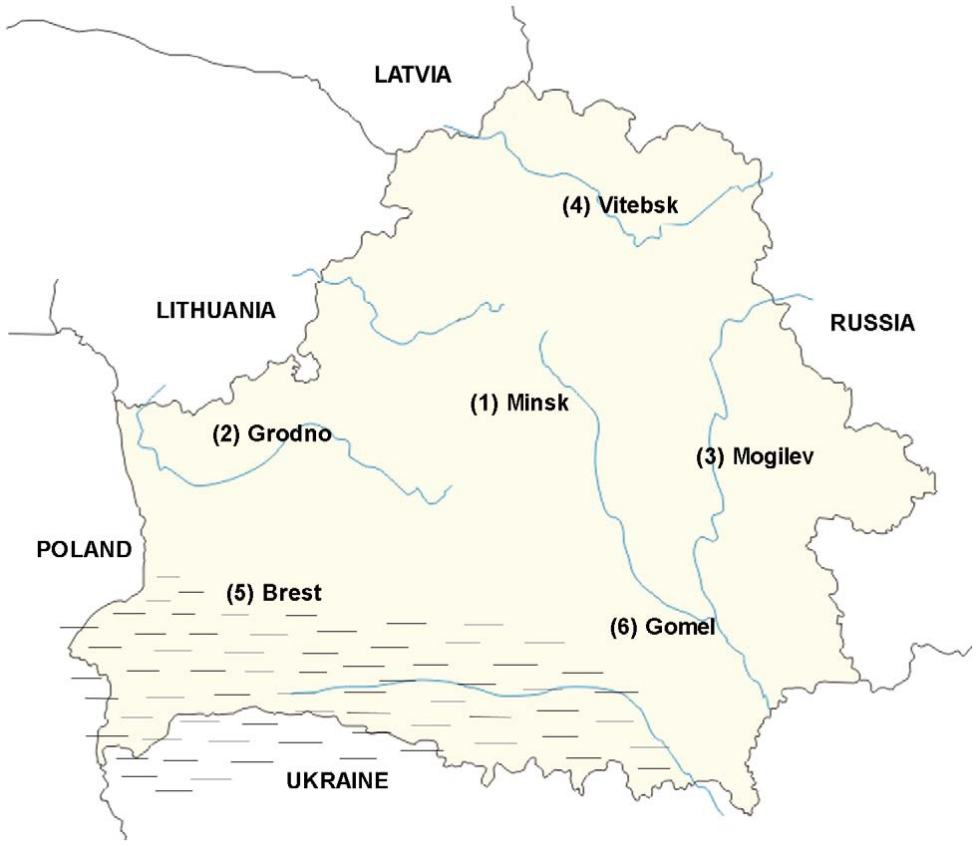
Map of Belarus. Figures indicate the regions where the population samples have been collected; the Polessie region is shown dashed.

The number of persons genotyped in six regions of Belarus (Figure 1) for estimation of the deafness major mutation carrier rate is presented in table 1. The mutation was found in 43 out of 757 persons with normal hearing, i.e. 5.7% - a high rate of incidence. We have found three regions of Belarus with an extremely high carrier rate: the south-west of the country (west Polessie) where it equals 10.2%, the northern part of Belarus (Vitebsk region) −6.1% and south-east (east Polessie) −5.9%. The northern part of Belarus is geographically proximal to Estonia, where the carrier rate of *35delG* was reported to be 4.5% [3]. Counter to this, in the western part of Belarus a carrier rate of 2% was revealed that is comparable to the mean European frequency for this mutation. The differences between *35delG* carrier rate in west Polessie and other regions of the country are therefore significant.

**Table 1:** The rate of *35delG* heterozygotes in the population cohort (native inhabitants from six regions of Belarus).

Belarus regions	Detected *35delG* heterozygotes/number of persons genotyped (%)
1 - center	5/126 (4.0)
2 - west	2/97 (2.0)
3 - east	5/127 (3.9)
**4 - north**	**8/131 (6.1)**
**5 - south-west**	**16/157 (10.2)** **
**6 - south-east**	**7/119 (5.9)**
Total	43/757 (5.7)

** The difference in rates between south-west and all the other regions is significant (P<0,02).

According to the results obtained, the map of the European distribution of *35delG* carrier frequencies [23] should be corrected since Belarusians are the population with the highest carrier rate so far reported, not only for Europe, but for the other parts of the world as well.

**Figure 2:**
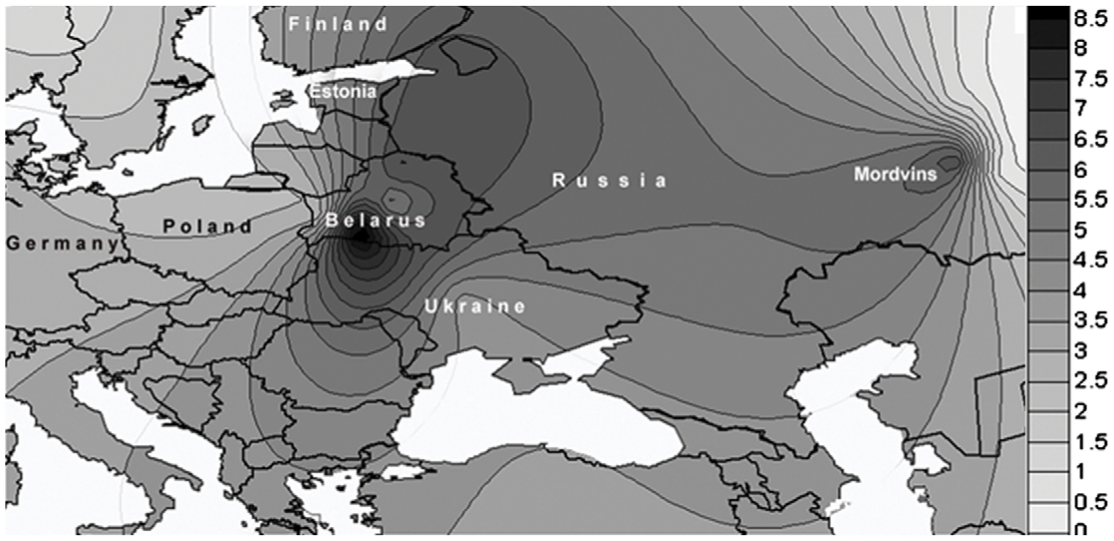
Geographic distribution of *35delG* carrier rate. The spatial frequency map was obtained by plotting the incidence of the *35delG* allele in 4346 individuals representing 20 populations including 6 sub-populations from Belarus together with others taken from the literature [12], [13]. The frequency data were converted to the isofrequency map using Surfer software (version 7, Golden Software Inc., Golden, CO, USA). The right panel shows how the color scale corresponds to *35delG* allele frequency (%).

The high *35delG* carrier rate in the Belarus population as a whole, together with the uneven distribution of the mutation carrier rate, seems surprising for this small country having no geographically-isolated zones. Some previous studies have assumed that the high frequency of the *35delG* mutation reflects the presence of a mutational hot spot [24], [25], while others support the theory of a common founder [26], [27]. Greece is among the countries with the highest carrier frequency of the mutation (3.5%) and, according to the idea of a common founder, the *35delG* appeared about 10000 years ago in the territory of contemporary Greece and expanded throughout Europe [28], [29]. The alternative model that a high frequency of the *35delG* mutation reflects the presence of a mutational hot spot seems a more probable explanation of the results obtained here. To prove or reject any of these hypotheses, single nucleotide polymorphisms adjacent to GJB2 (haplotypes) should be analyzed and genotyping of the native inhabitants from nearby Ukrainian and Polish Polessie and the regions of Russia adjoining the northern part of Belarus should be performed. A high *35delG* carrier frequency (6.2%) was reported recently for Mordvins, a small ethnic group that belongs to the Finno-Ugric linguistic group and inhabits the Volga-middle Ural region of Russia [13]. Taken together, Belarus as well as Estonia and Mordovia are three European regions with a *35delG* carrier frequency almost three times higher than that generally found in Europe and twice as high as that found in south-western Europe and it would be fascinating to know whether these three peaks are unrelated or have “common history" (Figure 2).

Data on the selective advantage of heterozygotes [14] should be taken into account not only to explain the high rate of *35delG* mutation among Belarusians but also to interpret its uneven distribution within the country depending on various ecological conditions and the resulting unevenness in the distribution of skin pathogens. Belarus Polessie (especially its western part, that was found to have the highest carrier rate of *35delG* mutation) is situated in the south of Belarus where the humidity is extremely high due to numerous swamps and dense woods, creating a favorable environment for skin pathogens (Figure 1).

### Analysis of the Belarus patient cohort with SNHL

#### 35delG mutation

All 391 patients with SNHL were screened for the 35delG GJB2 mutation. 178 patients (45.5%) were homozygous for this genetic defect, 51 (13.1%) had only one mutant allele and in 162 (41.4%) patients the major GJB2 mutation was not found (Table 2). Thus, as for other European SNHL patient cohorts, the 35delG point deletion is the major cause of prelingual SNHL among the Belarus patients studied.

**Table 2:** Genotype/phenotype correlations for the patients with (D) and without (N) *35delG* mutation.

SNHL degree	Patients analyzed	DD	DN	NN	%DD/%NN
moderate	18	3	2	13	16.7/72.2**
moderate/severe	23	7	1	15	30.4/65.2
severe	88	36	11	41	40.9/46.6
severe/profound	45	27	7	11	60.0/24.4
profound	217	105	30	82	48.4/37.8
Total	391	178	51	162	45.5/41.4

** The difference of DD % with other patient groups is significant (P<0,02).

**Figure 3:**
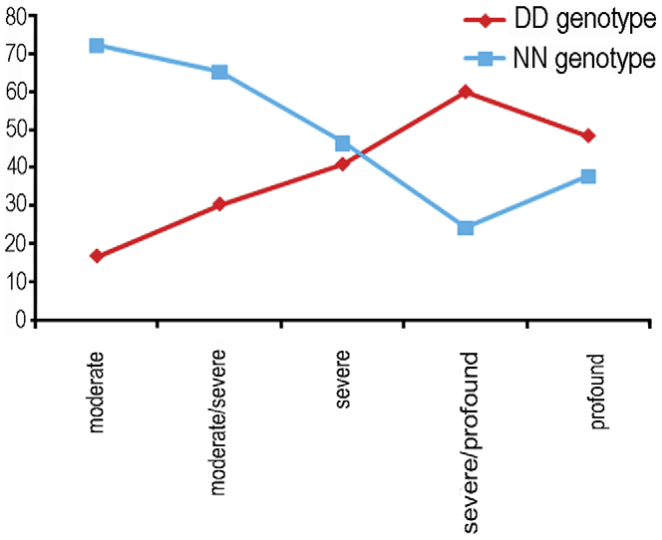
Percentages of DD and NN *35delG* genotypes among Belarus SNHL patients with different degrees of hearing loss.

The degree of SNHL varies among the studied Belarus patient cohort from moderate to severe (five grades in total). Among patients with moderate degree of hearing loss (HL) 72.2% lack the mutation and only 16.7% have *35delG/35delG* genotype whereas among the patients with severe HL 36 out of 88 (40,9%) were *35delG* homozygotes, with severe/profound HL 27 out of 45 (60%) and with profound HL 105 out of 217 (48,4%) (Table 2, Figure 3). Only 3 out of 178 homozygous *35delG* patients have moderate HL while 105 are totally deaf. Thus, based on the revealed phenotype-genotype association we conclude that among the Belarus patient cohort the *35delG/35delG* genotype results mainly in severe and profound hearing loss, which is similar to the other ethnic groups previously studied [5], [30].

#### Other mutations of the GJB2 gene and the del(GJB6-D13S1830)

SSCP analysis of the GJB2 coding exon was carried out for all DNA samples heterozygous or lacking the 35delG mutation. The samples with dissimilar mobility patterns were sequenced. All the patients except 35delG homozygotes were screened for the presence of GJB6 309 kb deletion: del(GJB6-D13S1830). The results are summarized in table 3.

**Table 3:** The rates of GJB2 and *del*(GJB6- D13S1830) mutations in Belarus SNHL patient cohort.

*GJB2* genotype	Number of mutation carriers in patient cohort (%)	Range of SNHL degree*
*35delG/35delG*	178 (45.5)	3 mod, 7 mod/s, 35 s, 27 s/pr, 106 pr
*35delG/312del14*	20 (5.1)	2 mod, 1 mod/s, 3 s, 3 s/pr, 8 pr
*N/312del14*	5 (1.28)	mod/s, s, s/pr, 2 pr
*35delG/del(GJB6-D13S1830)*	3 (0.77)	pr
*312del14/312del14*	2 (0.51)	pr
*35delG/235delC*	3 (0.77)	s
*167delT/235delC*	2 (0.51)	1 s, 1 s/pr
*35delG/I82M*	2 (0.51)	1 mod, 1x
*35delG/167delT*	2 (0.51)	1 s, 1 s/pr
*N/167delT*	2 (0.51)	1 pr, 1 s/pr
*N/V27I*	1 (0.26)	pr
*35delG/V27I+E114G*	1 (0.26)	s
*35delG/N*	20 (5.1)	
*Total*	241 (61.6)	

* Degree of HL: mod – moderate; s – severe, pr – profound, x - no audiological data.

For 31 out of 51 patients bearing one *35delG* allele, either another mutation in GJB2 gene or the 309 kb deletion *(GJB6-D13S1830)* was found; for the remaining 20 patients, only *35delG* was detected. According to our data, the second most common GJB2 mutation causing SNHL in Belarus patient cohort is *312del14*. We have detected 27 individuals bearing this mutation: two patients without 35*delG* were homozygous for *312del14,* 20 were *35delG/312del14* heterozygotes, and 5 patients lacking 35*delG* had one deleted *312del14* allele. The overall *312del14* allele frequency among SNHL patients is 3.7%. Six other single nucleotide deletions or substitutions previously reported to cause SNHL [3], [5], [31] were found in the Belarus patient cohort, all of them with a frequency of less than 1% (0.77%–0.26%) (Table 3). Notably, among 241 patients with SNHL that bear various GJB2 mutations, only four individuals were found with nucleotide substitutions, while the vast majority bear deletions: single-nucleotide (207 patients), deletion of 14 nucleotides (27 patients) and 309 kb (3 patients).

#### Mitochondrial mutations in population and patient cohorts

Two mitochondrial mutations, T961G and A1555G (both are located in mt-RNR1), were screened in the sample of 300 normal hearing ethnic Belarusians. 595 individuals with normal hearing were genotyped for the 7445/7444 (mt-TS1) mutations. All individuals from the patient cohort were genotyped for the four mtDNA mutations. The results are presented in table 4.

**Table 4:** Rates of mitochondrial mutations in the Belarus population and patient cohorts with SNHL.

	Mitochondrial mutation *(%)*			
	*T961G MT-RNR1*	*A1555G MT-RNR1*	*G7444A MT-TS1*	*A7445G MT-TS1*
Population Detected/studied	3/300 (1.0)	0/300 (0)	6/595 (1.01)	0/595 (0.0)
Patients Detected/studied	6/391 (1.5)	2/391 (0.51)	3/391 (0.77)	0/391 (0.0)

No individuals from the population cohort bear the *A1555G* mutation. Two patients with hearing loss possess this mitochondrial mutation (0.53%). Surprisingly, this rate is similar to that found among 513 Greek children with hearing loss (0.4%) [32] but is much less than the 3.6% found among SNHL patients of neighboring Poland [33].

Two other mitochondrial mutations were found both in the population and patient groups with rates 0.77–1.5%. There are various opinions concerning *T961G* mutation: it is often considered as a non-pathogenic polymorphism, but there are reports about its association with aminoglycoside-induced deafness and SNHL [19]. 6 representatives of our patient cohort have *T961G* (1.5%), five of them are also *35delG* homozygotes with a profound (4 persons) or severe (1 person) degree of hearing loss and one of these 6 patients has a non-mutant coding exon 2 of the GJB2 gene. Thus, the cause of deafness in these five patients is probably the *35delG* GJB2 pathogenic mutation, rather than the mitochondrial *T961G.* In the population cohort, *T961G* substitution was found in 3 persons out of 300 analyzed individuals (1%), and all of them still have normal hearing. Taking into consideration these results we suppose that *T961G* is either a non-pathogenic polymorphism, or possibly a pathogenic mutation with extremely low penetrance among the Belarusian patients cohort studied.

The pathogenic role of *G7444A* is also questionable. It is considered to be defining for mitochondrial haplogroup V [34], but its role in aggravating the expression of the *A1555G* mutation was also proposed [35]. *G7444A* incidence in the Belarusian population and patient cohorts studied is about 1% (Table 4). A high carrier rate of this mutation (1/62 or 1.6%) was found in Poland and as a result this substitution was suggested to be a nonpathogenic polymorphism in the Polish population [33]. In the present study, three patients with this substitution do not bear any mutation in either *GJB2* or in the mt*-RNR1* mitochondrial gene, and the genetic origin of their profound hearing loss remains uncertain.

In summary, the results from genotyping the large Belarus population cohort for the *35delG* mutation proves that the frequency of this mutation (5.7%) is the highest among all European countries studied. In one of the Belarus regions (west Polessie) the mutation frequency exceeds 10%. We show that the same single-nucleotide deletion, *35delG* in GJB2, is also the main genetic cause of *SNHL* in Belarus as it is detected in the homo- or heterozygous state in 53% of the patients with moderate-to-profound hearing loss. The higher the degree of hearing loss the higher the proportion of *35delG* homozygotes detected. Six other mutations in the GJB2 gene were found: *312del14* in 7% of patients and the rest in less than 1.5% of patients. The mitochondrial *A1555G* mutation was revealed in less than 1% of patients. No patient with *A7445G* was detected but three (0.77%) were found to carry *G7444A* substitution. We note that the rate of *G7444A* substitution in the population cohort is about 1%, evidence of its non-pathogenic state among Belarusians.

## Materials and Methods

### Ethics Statement

Both population and patient samples reported in the study were collected after obtaining informed written consent; written informed consent was obtained from the parents of the minors who took part in this study. The study has been considered and approved specifically by the Bioethics Committee of the Belarusian State Medical University (Minsk, Belarus) and the Scientific Board of the Institute of Genetics and Cytology of National Academy of Sciences (Minsk, Belarus).

The research consisted of two parts as follows:

1. Analysis of the carrier rate of *35delG* GJB2 mutation, as well as mtDNA mutations among native Belarusian people with normal hearing (referred to as the “population cohort" in the main text)
2. Detection of *35delG* GJB2, del(GJB6-D13S1830) deletions, revealing other structural changes in the GJB2 gene along with analysis of mtDNA mutations in the cohort of Belarus patients with SNHL (referred to as the “patient cohort" in the main text).

### Subjects under study

#### Part 1- Population cohort study

To characterize the Belarusian population, the country (9.5 million inhabitants) was divided into six sampling regions according to the ethno geographic approach; samples were collected in 2–4 small towns from each region (Figure 1). Blood samples from 757 native inhabitants of Belarus were collected after obtaining written informed consent. Each person filled in the questionnaire concerning his (her) ethnicity and place of birth, as well as ethnicity and place of birth of his (her) parents and grandparents. Only those volunteers who had normal hearing and lived in the particular region of the country for three or more generations were included in the study.

#### Part 2- Patient cohort study

391 young patients, mostly school- and preschool- children with sensorineural hearing loss (non-relatives, aged 1–25) with or without family history of SNHL, permanently living in Belarus, were taken into analysis after obtaining a written consent to be involved into the study from their parents or from themselves for adults. All hearing impaired patients have passed otoscopic examination and different audiological tests depending on their age: pure-tone audiometry, impedance audiometry, distortion-product otoacoustic emission, brainstem evoked response audiometry. Only those with bilateral hearing loss were included in the study. Additionally, all patients were assigned to groups according to the degree of SNHL (from moderate to profound) based on the audiological tests. We note that the patient cohort samples have been collected in the same six regions of the country as the population cohort samples.

#### DNA

For the population cohort study 5 ml of venous blood was collected, whereas 50–100 mcl (1–2 drops) of peripheral blood was collected for patients. DNA was isolated with proteinase K and phenol-chloroform purification [36].

### Strategy of genotyping

1) Population cohort study: the *35delG* GJB2 mutation was genotyped in 757 samples over six regions of Belarus. Smaller sample sets (while still representing all the regions) were analyzed for the presence of mitochondrial mutations. In particular, 300 samples were screened for mt-RNR1 mutations while mt-TS1 mutations were genotyped among 595 samples.

2) Patients cohort study

a) GJB genes: the *35delG* GJB2 mutation was analyzed for all patients with SNHL. Further, for the patients without the *35delG* mutation or heterozygous individuals, the SSCP technique was used to detect the structural changes in the coding part of the GJB2 gene. Direct sequencing of dissimilar patterns was used to specify the nucleotide sequence change. All the patients except the *35delG* homozygotes were screened for the 309 kb deletion: *del(GJB6-D13S1830)* using a multiple PCR protocol [37].

b) Mitochondrial mutations *A1555G, T961G, A7445G* and *G7444A* were detected after amplification with specific primers and cleavage by endonucleases, all mitochondrial mutations found were also confirmed by sequencing. See table 5 for all primers and conditions of genotyping.

**Table 5:** Primers, PCR conditions and endonucleases for genotyping.

*Gene*	*Mutation*	*Primers (nucleotide sequence or range)*	*Ta*	*Product size*	*Enzyme*	*Fragment size*		*Reference*
						*Norm*	*Mut*	
GJB2	*35delG*	*F gctggtggagtgtttgttcacacccgc*	*60^0^*	89	*MvaI*	60; 29	89	[38]
		*R tcttttccagagcaaacggc*						
309 kb deletion	*del(GJB6-D13S1830)*	*F1 gccatgcatgtggcctacta*	*58^0^*	480, 441	*-*	480	441	[37]
		*F2 cattgttgtgaactaacctcca*						
		*R1 actatctgaaatcagctcattc*						
mt-RNR1	*A1555G*	*F 1009–1032*	*55^0^*	566	*HaeIII*	455; 111	455; 91;20	[39]
		*R 1575–1556*						
mt-RNR1	*T961G*	*F 592–613*	*53^0^*	544	*AciI*	306; 246; 2	306; 186;60;2	*
		*R 1164–1145*						
mt-TS1**	*A7445G/G7445A*	*F 7402–7420*	*62^0^*	266	*XbaI*	226;40	266	*
		*R 7667–7649*						

Notes

* - self-designed primers;

** **-** for each sample without *XbaI* recognition site sequencing was carried out; Norm – normal, Mut – mutant alleles; the nucleotide positions and substitutions of mtDNA are given relative to the rCRS [40].
